# Transcriptome Analysis Reveals the Venom Genes of the Ectoparasitoid *Habrobracon hebetor* (Hymenoptera: Braconidae)

**DOI:** 10.3390/insects15060426

**Published:** 2024-06-05

**Authors:** Hongyan Yang, Jingyi Lu, Kui Wang, Chaoyan Wu, Bin Yang, Jiaying Zhu

**Affiliations:** 1 Key Laboratory of Forest Disaster Warning and Control of Yunnan Province, Southwest Forestry University, Kunming 650224, China; hongyanyang_yy@163.com (H.Y.); lujingyi@swfu.edu.cn (J.L.); wk7708@yeah.net (K.W.); wcy1316033@swfu.edu.cn (C.W.); yangbin48053@163.com (B.Y.); 2 Key Laboratory for Forest Resources Conservation and Utilization in the Southwest Mountains of China, Ministry of Education, Southwest Forestry University, Kunming 650224, China

**Keywords:** parasitoid, venom, gene expression, paralytic protein

## Abstract

**Simple Summary:**

The ectoparasitoid *Habrobracon hebetor* (Hymenoptera: Braconidae) exhibits broad parasitic abilities against lepidopteran pests, utilizing its venom as a key factor for host mortality. Analyzing the venom’s constituents is crucial to understanding the mechanisms of efficient host killing and identifying functional venom proteins. Transcriptomic analysis identified 34 venom proteins in *H. hebetor*, including serine protease, metalloproteinase, and esterase, as well as unique proteins like paralytic protein and ion transport peptide-like. Spatial gene expression profiling confirmed high expression of these venom proteins in the venom apparatus, particularly noting the importance of the paralytic protein in successful parasitism. This discovery of venom proteins sets the stage for research on bioactive agents for pest control.

**Abstract:**

The ectoparasitoid *Habrobracon hebetor* (Hymenoptera: Braconidae) exhibits a broad parasitic capability towards various lepidopteran pests, with venom serving as a crucial virulent factor ensuring successful parasitization and subsequent host mortality. Analyzing the constituents of its venom is essential for elucidating the mechanisms underlying efficient host killing by this parasitoid and for exploring potentially functional venom proteins. Through a transcriptomic analysis, a total of 34 venom proteins were identified within the venom of *H. hebetor*, encompassing known components such as serine protease, metalloproteinase, esterase, and serine protease inhibitors commonly present in parasitoid venoms. Unique components like paralytic protein and ion transport peptide-like were identified, possibly specific to certain parasitoids, along with novel proteins with uncharacterized functions. Spatial gene expression profiling of the identified venom proteins using transcriptomic data, corroborated by quantitative PCR validation for 13 randomly selected proteins, revealed abundant expression levels in the venom apparatus, affirming them as genuine venom components. Notably, the paralytic protein exhibited prominent expression, with the highest FPKM (fragments per kilobase of transcript per million fragments mapped) value of 24,704.87 in the venom apparatus, indicative of its significant role in successful parasitism by *H. hebetor*. The identification of these venom proteins establishes a foundation for the further exploration of bioactive agents for pest management strategies.

## 1. Introduction

Parasitoid wasps represent an exceedingly diverse group of insects that play a crucial role as natural enemies in the biological control of insect pest populations. They are classified into 12 superfamilies, encompassing 51–59 families [1,2]. Remarkably, they are estimated to comprise approximately 10% of all documented species on Earth, with conservative estimates placing their global species count between 500,000 and over 1 million [2]. Venom serves as a pivotal virulent factor employed by parasitoids to manipulate their hosts, creating a conducive environment for the successful development of their offspring [3,4]. Extensive research has demonstrated that parasitoid venom exhibits versatile biological functions, including the paralysis of hosts, regulation of the host’s development, suppression of the host’s immune response, and modulation of the host’s metabolism [5,6]. Owing to these unique functions, parasitoid venoms represent valuable resources for the discovery of bioactive molecules applicable in agricultural and medical sectors [7]. Unfortunately, only a small fraction of parasitoid species have been studied in order to elucidate their venom constituents [3,4,5,6,7,8]. A comprehensive understanding of venom components in a broader range of parasitoid species would greatly facilitate the comprehension of venom evolution and the construction of diverse venom gene repositories, ultimately enabling the identification of potent bioactive agents for pest control and other applications.

As one of the cosmopolitan parasitoids within the Hymenoptera family Braconidae, *Habrobracon hebetor* (Say), formerly known as *Bracon hebetor* Say, *Bracon juglandis* Ashmead, and *Hebrabracon junglandis* Ashmead, stands out as a gregarious idiobiont ectoparasitoid [9]. It is commonly found in stored grain ecosystems, where it plays a crucial role as a natural enemy of pyralid moths [10,11]. In addition, this parasitoid is known to attack certain stored product beetles and lepidopterous pests in agricultural fields [12,13]. Due to the ease of mass-rearing, a short developmental period during its immature stage, and its remarkable reproductive ability, *H. hebetor* has proven effective in managing populations of multiple lepidopteran pests in both stored products and field crops [14]. Notable examples documented in the literature include the Indian meal moth, *Plodia interpunctella*, a very pervasive household pest causing severe infestations in various stored and processed food products; the millet head miner moth, *Heliocheilus albipunctella*, a significant hindrance to millet production in Africa; and the cotton bollworm, *Helicoverpa armigera*, a widely distributed polyphagous pest that infests numerous agricultural crops worldwide [15,16,17]. Previous research on *H. hebetor* has predominantly revolved around its applications in biological control and various aspects of its biology [18,19,20,21]. However, our understanding of how it manipulates its host during parasitism remains limited. Investigations in this area will establish the groundwork for the successful utilization of this important biological agent.

Female *H. hebetor* adults display a preference for parasitizing the last instar larvae of their host species [22]. They achieve this by injecting venom into the larvae, rendering them paralyzed, before laying their eggs either directly on the immobilized larvae or in close proximity [23]. The immobilized host larvae then function as a nourishing food source for the growth and development of wasp offspring, and can serve as sustenance for adult females as well [22,24,25]. The paralytic effect of *H. hebetor* proves fatal to its hosts and can even result in the hosts’ rapid deaths within as little as 15 min [12,26]. Venom of *H. hebetor*, or its envenomation, has been shown to play a crucial role in manipulating the physiological processes of paralyzed host larvae. This includes actions such as blocking neuromuscular transmission [27,28,29]; changing the level of adipokinetic hormone [30,31]; inducing the expression of stress- and immune-associated genes [32,33]; inhibiting eicosanoid biosynthesis, leading to increased oxidative stress [34]; altering the midgut bacterial community [35]; and suppressing immunity through the inhibition of phenoloxidase activity, as well as a reduction in hemocyte encapsulation [36]. In addition, venom of *H. hebetor* has been found to exhibit anti-inflammatory properties by inhibiting nitric oxide production and reducing the levels of proinflammatory mediators and cytokines through modulation of the nuclear factor kappa B and mitogen-activated protein kinase pathways [37].

These findings imply the presence of active molecules in the venom of *H. hebetor* that serve multiple functions, supporting their potential utility in pest control. Although several studies have attempted to elucidate all the active venom components of *H. hebetor*, only one protein, weighing approximately 18 kDa, has been determined to act upon glutamatergic neuromuscular synapses [38]. Additionally, two partially purified venom proteins with molecular weights of approximately 43.7 kDa and 56.7 kDa, called A-MTX and B-MTX, have been implicated in the paralyzing activity [39]. Furthermore, two purified toxins known as Brh-I and Brh-V, each with an apparent molecular mass of 73 kDa, have demonstrated notable insecticidal properties. It is worth noting that among these venom proteins, only the latter has been partially sequenced, while the sequences of the others remain unknown [40,41]. Deciphering the venom components of this parasitoid will aid in elucidating how it utilizes venom to manipulate host physiology. This knowledge can potentially lead to the discovery of active molecules for the development of insecticidal products, which can be employed in the management of pest populations. In this study, we employed a transcriptomic approach to unveil the venom genes of *H. hebetor*, followed by validation of their expression profiles in various tissues of female adults. Herein, we present our findings.

## 2. Materials and Methods

### 2.1. Insects

The *H. hebetor* colony was initiated by sampling adults from the stock culture of the rice meal moth, *Corcyra cephalonica*. The rice meal moth population was sourced from stored rice that was previously infested by this pest. The infested rice was obtained from a farmer who had cultivated the rice in a suburban field situated in Kunming, China. Larvae of *C. cephalonica* were reared in our lab, in a plastic container where they consumed a diet composed of corn meal, soybean meal, and wheat bran in a 7:2:1 weight ratio. Adults of *C. cephalonica* were collected post-emergence and housed in a plexiglass cage that harbored a small glass container, which served as an egg-laying site. Eggs laid by the moths were gathered and relocated to a Petri dish filled with an artificial diet to nourish the hatching larvae. The last instar larvae of *C. cephalonica* were then used as hosts for the *H. hebetor*. The adult *H. hebetor*, with a male to female ratio of 1:1, were held in a 500 mL conical flask and provided with a 10% honey solution for food. All insects were maintained under controlled conditions of 25 ± 1 °C, 75 ± 5% relative humidity, and a 14 h light and 10 h dark photoperiod.

### 2.2. Transcriptome Sequencing

The venom apparatuses and residual bodies of 100 adult female *H. hebetor* were dissected in PBS on ice under a stereomicroscope. In addition, adult males were collected for analysis. Total RNA was extracted from these samples using TRIzol reagent (Invitrogen, Carlsbad, CA, USA) following the recommended manufacturer’s protocol. The content, quality, and integrity of the total RNA were determined using a Nanodrop spectrophotometer (IMPLEN, Westlake Village, CA, USA), 1% agarose gel electrophoresis, and an Agilent 5400 Fragment Analyzer system (Agilent Technologies, Santa Clara, CA, USA), respectively. mRNA was then enriched from the total RNA using magnetic beads with attached Oligo(dT). This mRNA was subsequently used for cDNA library construction, which involved the use of the TruSeq RNA Sample Prep Kit (Illumina Inc., San Diego, CA, USA), as guided by the manufacturer’s instructions. The libraries were then sequenced using the Illumina HiSeq 2500 platform (Illumina Inc., San Diego, CA, USA). Three distinct libraries were constructed and subjected to sequencing for each sample analyzed. The generated raw data have been deposited into the National Center for Biotechnology Information (NCBI)’s Sequence Read Archive under BioProject accession PRJNA1114022. After the raw data underwent a quality control process, they were used to remove the adapters and low-quality reads through Trimmomatic v1.4 [42] with its default parameters. The resulting clean data were assembled into transcripts with Trinity v2.8.5 [43]. Subsequently, these transcripts were used to generate unigenes with the assistance of Corset v1.09 (https://github.com/Oshlack/Corset/wiki, accessed on 20 July 2023) [44]. The completeness of the transcriptome was evaluated through the employment of the bioinformatic software BUSCO v5.3.2 (http://busco.ezlab.org, accessed on 16 May 2022).

### 2.3. Identification of Venom Proteins

The clean reads from three samples were mapped onto the assembled unigenes using the Kallisto Super Wrapper function in TBtools v1.0987663 [45]. Following this process, the read count figures for each gene from the individual samples were utilized in the calculation of the FPKM (fragments per kilobase of transcript per million fragments mapped) values. Differential gene expression analyses between various samples were performed using DEseq2 v1.40.2 [46]. The identification process of differentially expressed genes within the venom apparatus, compared to those in the residual body, used a specified criteria of a significant adjusted *p*-value of less than 0.05 and an absolute fold change greater than 1.5. The proteins encoded by these differentially expressed genes (restricted to those appearing in the top 500 by FPKM) were searched for the presence of a signal peptide. Proteins possessing the signal peptide were considered venom protein candidates. For genes lacking a complete open reading frame and missing the N-terminus, homologous sequences showing the highest similarity in the NCBI nr database were retrieved for signal peptide prediction. If these homologous sequences contained a signal peptide, the respective genes were classified as encoding secreted proteins. Candidate proteins were then screened further to exclude housekeeping proteins following the criteria delineated by Walker et al. [47]. The final identified venom proteins were functionally annotated by performing a BLASTP v2.10.1 search against the NCBI nr database.

### 2.4. Expression Profiling of Venom Genes

The FPKM values for the identified venom genes were obtained from three independent biological replicates of the venom apparatuses and residual bodies of the female adults, as well as the male adults. These values were then used to illustrate their expression patterns in a heatmap using TBtools v1.0987663 [45]. Moreover, certain venom genes were selected for further validation via quantitative real-time PCR (qPCR). The extraction of the total RNAs from the venom apparatuses and residual bodies of the female adults, as well as the male adults, was conducted as described above. The gene sequences of venom proteins were retrieved from the previously generated transcriptomic data. Gene-specific primers, according to their gene sequences, were designed using Beacon Designer 8.14 software (PREMIER Biosoft International, Palo Alto, CA, USA) (Table S1). The qPCR was performed utilizing the Bestar^®^ Sybr Green qPCR Master Mix (DBI Bioscience, Shanghai, China) under the following conditions: an initial pre-denaturation at 95 °C for 2 min, followed by 95 °C for 10 s, 58 °C for 31 s, and 72 °C for 30 s over the course of 40 cycles. This was conducted using a qTOWER 2.2 Real Time qPCR Thermal Cycler (Analytik Jena AG, Jena, Germany). Each sample included three biological duplicates, and each duplicate had three technical repeats. The data for each replicate were derived from the average of three technical repeats. Subsequently, the mean value obtained from the three biological replicates for each sample was subjected to further analysis. Data interpretation was performed by employing the Q-gene method [48,49]. GraphPad Prism 8.0 (GraphPad Software Inc., San Diego, CA, USA) was used for statistical analysis, specifically via one-way analysis of variance (ANOVA) with a significance threshold of *p* < 0.05. The data were also visualized using GraphPad Prism 8.0.

## 3. Results

### 3.1. Transcriptome Sequencing and Assembly

Illumina sequencing yielded a total of 152,419,904, 181,800,400, and 173,007,912 raw reads for the venom apparatuses, the residual bodies of the female adults, and the male adults of *B. hebetor*, respectively (Table 1). Following quality control, clean data of 22.84 G, 27.22 G, and 25.90 G were obtained for these samples from their raw data. The data exhibited an error rate of 0.03% or lower, with Q20 values exceeding 96% and Q30 values surpassing 89%. The clean data were then assembled into 124,243 transcripts and 48,032 unigenes with mean lengths of 1351 bp and 1433 bp, respectively (Table 1). The unigenes were assessed in order to determine the completeness of the assembled transcriptome, yielding a complete BUSCO score of 97.50%, indicating a high level of transcriptomic assembly completeness.

### 3.2. Identification of Venom Proteins

Through a transcriptomic analysis, a total of 34 venom proteins were identified and categorized, based on their functional annotations, into five principal groups comprising proteases and peptidases; protease inhibitors; chaperones; other known proteins; and unknown proteins (Table 2). The most prevalent group among these proteins was proteases and peptidases (12), followed by proteins of unknown function (10). Specifically, within the proteases and peptidases category, the identified proteins included serine protease, metalloproteinase, lipase, esterase, cathepsin L, protein disulfide isomerase, and dolichyl-diphosphooligosaccharide-protein glycosyltransferase (DDOST). The identified protease inhibitors were classified as serine protease inhibitors. Aside from these venom proteins, the other identified proteins with recognized functions encompass heat shock protein 70, endoplasmin, paralytic protein, calreticulin, ion transport peptide-like, protein yellow, and juvenile hormone-binding protein.

### 3.3. Expression Patterns of Venom Genes

**Figure 1:**
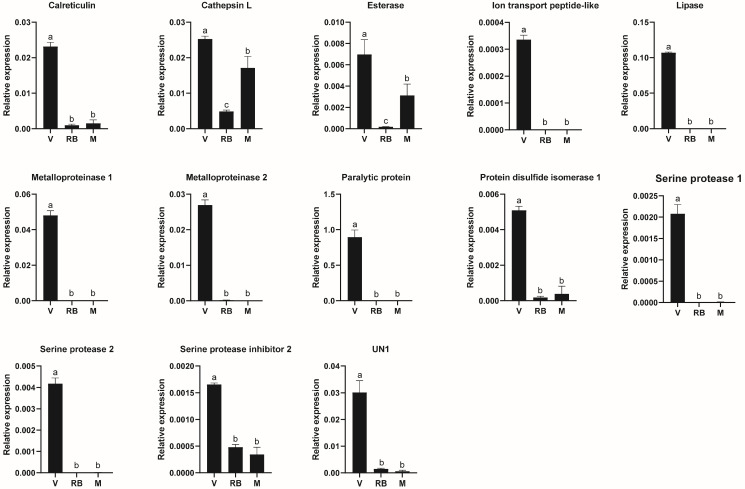
Expression profiles of venom genes of *Habrobracon hebetor*. V, venom apparatus; RB, residual body of female adult, female adult body deprived of venom apparatus; M, male adult. Significant differences (*p* < 0.05) are denoted using different letters above bars.

Utilizing the FPKM values, the expression levels of identified venom genes were profiled across the venom apparatuses, the residual bodies of female adults, and the male adults. The majority of these genes exhibited exclusive or pronounced expression in the venom apparatuses. Although the esterase, cathepsin L, and juvenile hormone-binding protein genes displayed high expression levels in both the venom apparatuses and the male adults, they showed notably higher expression levels in the venom apparatuses compared to the residual bodies of female adults. To validate the gene expression profiles inferred from FPKM values, a subset of 13 venom genes was selected for quantification using qPCR across the aforementioned tissues (Figure 1 and Table S2). The qPCR results demonstrated a strong concordance with the FPKM-derived expression patterns, attesting to the reliability of the transcriptomic profiling data. The paralytic protein emerged as the most abundantly expressed venom gene, as inferred from its FPKM values in the venom apparatus. Consistent with this, qPCR analysis revealed abundant and exclusive expression of the paralytic protein gene in the venom apparatus, indicating its prevalent presence in the venom of *H. hebetor*.

## 4. Discussion

In this study, stringent criteria established by Martinson et al. [50] were employed to identify authentic venom proteins in *H. hebetor*. Specifically, the criteria require the venom proteins to be highly expressed in the venom apparatus—with FPKM values ranking prominently among all genes of the species—and to exhibit secretion signals as indicated by the presence of signal peptides. A total of 34 venom proteins meeting these criteria were identified. In a previous study, the venom gland transcriptome of *B. hebetor*, synonymous to *H. hebetor*, was examined, revealing a substantial proportion of assembled contigs with strong similarity to those found in *Diachasma alloeum* [51]. Within this dataset, several venom components of *B. hebetor* were identified that exhibit high expression levels in its venom gland, such as acid phosphatase, arginine kinase, and venom allergen [51]. To clarify, the venom components identified in the previous study were not detected in the current research due to differences in the methodologies employed. In the previous study, Manzoor et al. [51] did not utilize a targeted approach to exclusively focus on venom genes that exhibit significantly higher expression levels in the venom apparatuses compared to the residual bodies of female adults. The rigorous methodology utilized in the current study accounts for the absence of these specific venom components in the collected dataset. In addition, a transcriptomic approach was employed to identify 152 venom proteins in the venom glands of *H. hebetor* [52]. A significantly larger number of venom proteins were identified in comparison to our current study. This disparity might be attributed to the implementation of a more stringent screening criterion in our research, following the method proposed by Martinson et al. [50], which involves filtering only the top 500 abundant transcripts in venom glands to identify potential venom genes. Furthermore, it is worth noting that different colonies of the same parasitoid species can exhibit significant genetic variability in their venom compositions. For instance, intraspecific venom variation has been identified in two strains of *Tetrastichus brontispae* [53]. One strain primarily parasitizes the *Brontispa longissima* pupa, while the other strain, a derivative of the former strain, has been sequentially exposed to the *Octodonta nipae* pupa as a host for over 40 generations [53].

The expressions of the venom proteins identified in this study were confirmed through qPCR, demonstrating abundant expression in the venom apparatus. These findings support the designation of these identified proteins as genuine venom components, warranting further investigation into their functional properties. Notably, while most of these proteins could be functionally categorized and are commonly observed in the venoms of parasitoids within the Braconidae family and other investigated families [54,55], DDOST and those venom proteins with unknown functions stood out as an exception. DDOST was recently recognized as a venom component in the venom of the assassin bug *Sycanus croceovittatus* [56]. Those venom proteins in *H. hebetor* that lack known functions may represent recently evolved components that warrant detailed functional elucidation.

Consistent with findings regarding the venom components of other parasitoids, proteases and peptidases, which make up 35.29% of all identified venom proteins, were found to be major constituents in the venom of *H. hebetor*. Among them, the serine protease homolog venom protein, identified as a venom component in all studied parasitoids, has been shown to play a critical role in suppressing hemolymph melanization in hosts by interfering with the phenoloxidase cascade in *Cotesia rubecula* and *Scleroderma guani* [57,58,59]. An ectoparasitic wasp, *Eulophus pennicornis*, possesses a venom metalloproteinase, EpMP3, which can impede the development and growth of its host, the tomato moth *Lacanobia oleracea* [60]. Likewise, a metalloprotease named VRF1, identified in the venom of the endoparasitoid wasp *Microplitis mediator*, possesses the capacity to disrupt the Toll signaling pathway in the hemocytes of its host, the cotton bollworm *Helicoverpa armigera*, thereby impacting the encapsulation of its eggs within the host [61]. The serine protease inhibitor, also referred to as a serpin, among the identified venom proteins has been revealed to possess the capacity to hinder its host’s prophenoloxidase activation and the synthesis of antimicrobial peptides in parasitoid venoms [62,63,64,65]. Apart from these three venom proteins, only one other venom protein, calreticulin, has demonstrated its ability to suppress the innate immune response to parasitoid venom, namely by modulating encapsulation reactions, and to decrease host bleeding during adult and larval parasitoid feeding [66,67,68]. The functions of the remaining venom proteins are yet to be clarified. It is possible that those venom proteins with unidentified annotated functions may have evolved distinct roles in *H. hebetor* to serve as crucial factors in manipulating its host organisms.

The whole venom of *H. hebetor* has been shown to participate in inhibiting the phenoloxidase activity and encapsulation rate in the larval hemolymph of the wax moth, *Galleria mellonella* [36]. These venom activities in *H. hebetor* are likely attributed to serine protease, serine protease inhibitor, metalloprotease, and calreticulin as venom constituents in this parasitoid, mirroring their roles observed in the venoms of other parasitoids as discussed earlier. The decreased hemocyte adhesion in *G. mellonella* larvae due to envenomation by the parasitoid *H. hebetor* is associated with elevated intracellular Ca^2+^ levels and increased phospholipase C activity in the host’s hemocytes [69]. Given the crucial involvement of calreticulin in insect cellular immune responses, the presence of calreticulin as a venom component in *H. hebetor* may significantly contribute to the suppression of the host’s cellular immunity [70,71]. Envenomation by *H. hebetor* can rapidly paralyze host larvae [72], triggering an increase in adipokinetic hormone levels in the central nervous systems of the cockroach *Periplaneta americana* and adult females of the firebug *Pyrrhocoris apterus* [30,31]. This elevation implies the involvement of adipokinetic hormone in mitigating the neuromuscular paralysis caused by *H. hebetor* venom. However, *H. hebetor* venom was found to have no inhibitory effect on the depolarization-dependent Ca^2+^ influx into the nerve terminal [29]. According to gene expression data, paralytic protein emerges as a prominent component in *H. hebetor* venom. The abundance of this venom protein likely plays a key role in effectively inducing paralysis in hosts of this parasitoid. The roles of the aforementioned venom proteins and other identified proteins within the venom of *H. hebetor* warrant further investigation through gene expression to produce recombinant proteins, enabling the exploration of their physiological roles. Moreover, recent advancements in genome editing using the CRISPR/Cas9 system [73] offer a valuable tool for elucidating the functions of *H. hebetor*’s venom proteins without compromising its basic biological functions.

## 5. Conclusions

In summary, this study identified 34 venom proteins in *H. hebetor*, shedding light on the mechanisms underlying its efficient host killing. The venom contains common components like serine protease, metalloproteinase, esterase, and serine protease inhibitors, along with unique components such as paralytic protein and ion transport peptide-like, potentially specific to certain parasitoids. Spatial gene expression profiling confirmed abundant expression of these venom proteins in the venom apparatus, particularly highlighting the significant role of the paralytic protein in successful parasitism. These findings not only enhance our understanding of the virulence mechanisms employed by this parasitoid, but also pave the way for further exploration of bioactive agents for pest management strategies.

## Figures and Tables

**Table 1:** Overview of *Habrobracon hebetor* transcriptome.

Total raw reads from venom apparatus	152,419,904
Total raw reads from residual body of female adult	181,800,400
Total raw reads from male adult	28,408,003
Total clean reads from venom apparatus	152,203,106
Total clean reads from residual body of female adult	181,466,566
Total clean reads from male adult	28,354,031
Total clean bases from venom apparatus (Gb)	22.84
Total clean bases from residual body of female adult (Gb)	27.22
Total clean bases from male adult (Gb)	25.90
Average Q20 (%)	97.23
Average Q30 (%)	92.51
Total number of transcripts	124,243
Total number of unigenes	48,032
Average length of transcripts (bp)	1351
Average length of unigenes (bp)	1433
N50 length of transcripts (bp)	3575
N50 length of unigenes (bp)	2792

**Table 2:** Venom proteins of *Habrobracon hebetor* identified through a transcriptomic approach.

Protein Name	V-FPKM	RB-FPKM	M-FPKM	Log2 Fold Change (V/RB)
Metalloproteinase 1	2810.63	5.60	1.29	9.50
Metalloproteinase 2	1096.56	3.00	1.78	9.02
Metalloproteinase 3	188.50	1.10	0.81	7.95
Lipase	2768.74	8.42	1.00	8.84
Esterase	297.07	37.61	244.31	3.58
Cathepsin L	618.41	391.25	542.74	1.65
Serine protease 1	308.50	1.31	0.55	8.47
Serine protease 2	264.87	0.66	0.67	9.39
Protein disulfide isomerase 1	1270.04	138.65	132.93	3.91
Protein disulfide isomerase 2	316.51	151.47	69.53	1.66
Protein disulfide isomerase 3	175.05	42.11	31.75	2.73
Dolichyl-diphosphooligosaccharide-protein glycosyltransferase	124.12	60.67	26.70	1.67
Serine protease inhibitor 1	756.02	115.33	73.18	3.38
Serine protease inhibitor 2	96.54	44.11	63.42	1.72
Serine protease inhibitor 3	95.25	32.18	20.70	2.14
Serine protease inhibitor 4	92.89	7.81	11.28	4.25
Heat shock protein 70a	535.27	54.63	77.51	3.99
Heat shock protein 70b	505.08	79.09	78.61	3.35
Endoplasmin	511.93	17.27	29.6	5.60
Paralytic protein	24,704.87	89.50	0.40	8.85
Calreticulin	1157.45	185.77	159.8	3.33
Ion transport peptide-like	1140.85	2.68	2.45	9.14
Protein yellow	304.70	13.85	11.37	5.12
Juvenile hormone-binding protein	120.57	44.71	90.3	2.00
UN 1	1560.57	611.29	751.49	1.84
UN 2	1389.84	350.48	103.49	2.65
UN 3	896.64	83.98	54.71	4.09
UN 4	356.29	7.10	6.66	6.37
UN 5	289.76	3.79	1.65	6.92
UN 6	223.28	8.66	11.26	5.46
UN 7	197.79	3.32	1.04	6.63
UN 8	137.39	19.82	29.31	3.37
UN 9	106.73	14.49	30.78	3.61
UN 10	103.53	14.64	11.80	3.45

V, venom apparatus; RB, residual body of female adult—female adult body deprived of venom apparatus; M, male adult.

## Data Availability

The sequencing data generated for this study have been deposited in the National Center for Biotechnology Information (NCBI)’s Sequence Read Archive (SRA), and can be accessed under the BioProject accession number PRJNA1114022.

## Supplementary Materials

The following supporting information can be downloaded at: https://www.mdpi.com/article/10.3390/insects15060426/s1, Table S1: Gene-specific primers used for qPCR analysis; Table S2: Fold changes of venom genes expressed in the venom apparatus compared to the female residual body, determined using qPCR.

## References

[1] Blaimer B.B., Santos B.F., Cruaud A., Gates M.W., Kula R.R., Mikó I., Rasplus J.Y., Smith D.R., Talamas E.J., Brady S.G. (2023). Key innovations and the diversification of Hymenoptera. Nat. Commun..

[2] Burke G.R., Sharanowski B.J. (2024). Parasitoid wasps. Curr. Biol..

[3] Poirié M., Colinet D., Gatti J.L. (2014). Insights into function and evolution of parasitoid wasp venoms. Curr. Opin. Insect Sci..

[4] Yang L., Qiu L.M., Fang Q., Stanley D.W., Ye G.Y. (2021). Cellular and humoral immune interactions between *Drosophila* and its parasitoids. Insect Sci..

[5] Yan Z., Fang Q., Song J., Yang L., Xiao S., Wang J., Ye G. (2023). A serpin gene from a parasitoid wasp disrupts host immunity and exhibits adaptive alternative splicing. PLoS Pathog..

[6] Russo E., Di Lelio I., Shi M., Becchimanzi A., Pennacchio F. (2023). *Aphidius ervi* venom regulates *Buchnera* contribution to host nutritional suitability. J. Insect Physiol..

[7] Asgari S., Rivers D.B. (2011). Venom proteins from endoparasitoid wasps and their role in host-parasite interactions. Annu. Rev. Entomol..

[8] Inwood S.N., Harrop T.W.R., Dearden P.K. (2023). The venom composition and parthenogenesis mechanism of the parasitoid wasp *Microctonus hyperodae*, a declining biocontrol agent. Insect Biochem. Mol. Biol..

[9] Krombein K.V., Hurd P.D., Smith D.R., Burks B.D. (1979). Catalog of Hymenoptera in America North of Mexico.

[10] Antolin M.F., Strand M.R. (1992). Mating system of *Bracon hebetor* (Hymenoptera: Braconidae). Ecol. Entomol..

[11] Baker J.E., Fabrick J.A. (2000). Host hemolymph proteins and protein digestion in larval *Habrobracon hebetor* (Hymenoptera: Braconidae). Insect Biochem. Mol. Biol..

[12] Cantori L.V., Garcia A.G., Pinto A.d.S., Godoy W.A.C., Parra J.R.P. (2022). Detailed look at paralysis of hosts by the ectoparasitoid *Habrobracon hebetor* (Hymenoptera: Braconidae): Does more efficient paralysis mean more effective parasitism?. BioControl.

[13] Abou El-Ela A.S., Dessoky E.S., Masry S., Arshad A., Munawar A., Qamer S., Abdelkhalek A., Behiry S.I., Kordy A. (2021). Plasticity in life features, parasitism and super-parasitism behavior of *Bracon hebetor*, an important natural enemy of *Galleria mellonella* and other lepidopteran host species. Saudi J. Biol. Sci..

[14] Chen H., Zhang H., Zhu K.Y., Throne J.E. (2012). Induction of reproductive diapause in *Habrobracon hebetor* (Hymenoptera: Braconidae) when reared at different photoperiods at low temperatures. Environ. Entomol..

[15] Jaafari-Behi V., Ziaee M., Kocheili F., Ali Hemmati S., Francikowski J. (2023). Life-table parameters of *Plodia interpunctella* (Lepidoptera: Pyralidae) on different stored date palm fruits under laboratory conditions. J. Insect Sci..

[16] Baoua I.B., Ba M.N., Amadou L., Kabore A., Dabire-Binso C.L. (2018). Field dispersal of the parasitoid wasp *Habrobracon hebetor* (Hymenoptera: Braconidae) following augmentative release against the millet head miner *Heliocheilus albipunctella* (Lepidoptera: Noctuidae) in the Sahel. Biocontrol Sci. Technol..

[17] Salehipour H., Vahedi H., Naghdeh N., Zamani A. (2017). Evaluation of population density and parasitism (Hubner) *Helicoverpa armigera* and *Spodoptera exigua* (Hubner) on twelve tomato cultivars under field conditions. J. Plant Prot..

[18] Ghimire M.N., Phillips T.W. (2010). Suitability of different lepidopteran host species for development of *Bracon hebetor* (Hymenoptera: Braconidae). Environ. Entomol..

[19] Howard R.W., Baker J.E. (2003). Cuticular hydrocarbons and wax esters of the ectoparasitoid *Habrobracon hebetor*: Ontogenetic, reproductive, and nutritional effects. Arch. Insect Biochem. Physiol..

[20] Mbata G.N., Warsi S. (2019). *Habrobracon hebetor* and *Pteromalus cerealellae* as tools in post-harvest integrated pest management. Insects.

[21] Yu K., Xiong S., Xu G., Ye X., Yao H., Wang F., Fang Q., Song Q., Ye G. (2020). Identification of neuropeptides and their receptors in the ectoparasitoid, *Habrobracon hebetor*. Front. Physiol..

[22] Akinkurolere R.O., Boyer S., Chen H., Zhang H. (2009). Parasitism and host-location preference in *Habrobracon hebetor* (Hymenoptera: Braconidae): Role of refuge, choice, and host instar. J. Econ. Entomol..

[23] Solà M., Castañé C., Lucas E., Riudavets J. (2018). Optimization of a banker box system to rear and release the parasitoid *Habrobracon hebetor* (Hymenoptera: Braconidae) for the control of stored-product moths. J. Econ. Entomol..

[24] Hagstrum D.W., Smittle B.J. (1978). Host utilization by *Bracon hebetor*. Environ. Entomol..

[25] Manishkumar R., Dhirubhai M., Piyushbhai R. (2013). Reproductive parameters of *Bracon hebetor* Say on seven different hosts. Afr. J. Agric. Res..

[26] Beard R.L., Connecticut Agricultural Experiment Station (1952). Toxicology of Habrobracon venom—A Study of a Natural Insecticide.

[27] Usherwood P.N.R., Machili P. (1966). Chemical transmission at the insect excitatory neuromuscular synapse. Nature.

[28] Walther C., Rathmayer W. (1974). The effect of *Habrobracon* venom on excitatory neuromuscular transmission in insects. J. Comp. Physiol..

[29] Walther C., Reinecke M. (1983). Block of synaptic vesicle exocytosis without block of Ca^2+^-influx. An ultrastructural analysis of the paralysing action of habrobracon venom on locust motor nerve terminals. Neuroscience.

[30] Shaik H.A., Mishra A., Kodrík D. (2017). Beneficial effect of adipokinetic hormone on neuromuscular paralysis in insect body elicited by braconid wasp venom. Comp. Biochem. Physiol..

[31] Karbusová N., Gautam U.K., Kodrík D. (2019). Effect of natural toxins and adipokinetic hormones on the activity of digestive enzymes in the midgut of the cockroach *Periplaneta americana*. Arch. Insect Biochem. Physiol..

[32] Shafeeq T., UlAbdin Z., Lee K.Y. (2017). Induction of stress- and immune-associated genes in the Indian meal moth *Plodia interpunctella* against envenomation by the ectoparasitoid *Bracon hebetor*. Arch. Insect Biochem. Physiol..

[33] Shim J.K., Ha D.M., Nho S.K., Song K.S., Lee K.Y. (2008). Upregulation of heat shock protein genes by envenomation of ectoparasitoid *Bracon hebetor* in larval host of Indian meal moth *Plodia interpunctella*. J. Invertebr. Pathol..

[34] Büyükgüzel E., Erdem M., Tunaz H., Küçük C., Atılgan U.C., Stanley D., Büyükgüzel K. (2017). Inhibition of eicosanoid signaling leads to increased lipid peroxidation in a host/parasitoid system. Comp. Biochem. Physiol..

[35] Polenogova O.V., Kabilov M.R., Tyurin M.V., Rotskaya U.N., Krivopalov A.V., Morozova V.V., Mozhaitseva K., Kryukova N.A., Alikina T., Kryukov V.Y. (2019). Parasitoid envenomation alters the *Galleria mellonella* midgut microbiota and immunity, thereby promoting fungal infection. Sci. Rep..

[36] Kryukova N.A., Dubovskiy I.M., Chertkova E.A., Vorontsova Y.L., Slepneva I.A., Glupov V.V. (2011). The effect of *Habrobracon hebetor* venom on the activity of the prophenoloxidase system, the generation of reactive oxygen species and encapsulation in the haemolymph of *Galleria mellonella* larvae. J. Insect Physiol..

[37] Saba E., Shafeeq T., Irfan M., Lee Y.Y., Kwon H.W., Seo M.G., Park S.-J., Lee K.Y., Rhee M.H. (2017). Anti-inflammatory activity of crude venom isolated from parasitoid wasp, *Bracon hebetor* Say. Mediat. Inflamm..

[38] Slavnova T., Antonov S., Magazanik L., Tonkikh A., Kosovskii A., Sadykov A., Abduvakhabov A. (1987). Effect of toxin from the venom of the ichneumon *Habrobracon hebetor* (Say) on neuromuscular transmission in insects. Dokl. Akad. Nauk SSSR.

[39] Visser B.J., Labruyère W.T., Spanjer W., Piek T. (1983). Characterization of two paralysing protein toxins (A-MTX and B-MTX), isolated from a homogenate of the wasp *Microbracon hebetor* (Say). Comp. Biochem. Physiol..

[40] Quistad G.B., Nguyen Q., Bernasconi P., Leisy D.J. (1994). Purification and characterization of insecticidal toxins from venom glands of the parasitic wasp, *Bracon hebetor*. Insect Biochem. Mol. Biol..

[41] Quistad G.B., Leisy D. (1997). Insecticidal toxins from the parastic wasp, *Bracon hebetor*. J. Clean. Prod..

[42] Bolger A.M., Lohse M., Usadel B. (2014). Trimmomatic: A flexible trimmer for Illumina sequence data. Bioinformatics.

[43] Grabherr M.G., Haas B.J., Yassour M., Levin J.Z., Thompson D.A., Amit I., Adiconis X., Fan L., Raychowdhury R., Zeng Q. (2011). Full-length transcriptome assembly from RNA-Seq data without a reference genome. Nat. Biotechnol..

[44] Davidson N.M., Oshlack A. (2014). Corset: Enabling differential gene expression analysis for de novo assembled transcriptomes. Genome Biol..

[45] Chen C., Chen H., Zhang Y., Thomas H.R., Frank M.H., He Y., Xia R. (2020). TBtools: An integrative toolkit developed for interactive analyses of big biological data. Mol. Plant.

[46] Love M.I., Huber W., Anders S. (2014). Moderated estimation of fold change and dispersion for RNA-seq data with DESeq2. Genome Biol..

[47] Walker A.A., Mayhew M.L., Jin J., Herzig V., Undheim E.A.B., Sombke A., Fry B.G., Meritt D.J., King G.F. (2018). The assassin bug *Pristhesancus plagipennis* produces two distinct venoms in separate gland lumens. Nat. Commun..

[48] Muller P., Janovjak H., Miserez A., Dobbie Z. (2002). Processing of gene expression data generated by quantitative real-time RT-PCR. Biotechniques.

[49] Simon P. (2003). Q-Gene: Processing quantitative real-time RT-PCR data. Bioinformatics.

[50] Martinson E.O., Kelkar Y.D., Chang C.H., Werren J.H. (2017). The evolution of venom by co-option of single-copy genes. Curr. Biol..

[51] Manzoor A., UlAbdin Z., Webb B.A., Arif M.J., Jamil A. (2016). De novo sequencing and transcriptome analysis of female venom glands of ectoparasitoid *Bracon hebetor* (Say.) (Hymenoptera: Braconidae). Comp. Biochem. Physiol..

[52] Yu K., Chen J., Bai X., Xiong S., Ye X., Yang Y., Yao H., Wang F., Fang Q., Song Q. (2023). Multi-omic identification of venom proteins collected from artificial hosts of a parasitoid wasp. Toxins.

[53] Tang B.Z., Meng E., Zhang H.J., Zhang X.M., Asgari S., Lin Y.P., Lin Y.Y., Peng Z.Q., Qiao T., Zhang X.F. (2019). Combination of label-free quantitative proteomics and transcriptomics reveals intraspecific venom variation between the two strains of *Tetrastichus brontispae*, a parasitoid of two invasive beetles. J. Proteom..

[54] Quicke D.L.J., Butcher B.A. (2021). Review of venoms of non-polydnavirus carrying ichneumonoid wasps. Biology.

[55] Li J., Zhou T., Zhu X., Wang L., Zhang K., Li D., Ji J., Luo J., Cui J., Gao X. (2024). Comparative transcriptome and proteome reveal the unique genes and proteins of female parasitic wasps, *Lysiphlebia japonica* Ashmead. Pest Manag. Sci..

[56] Wu C., Li L., Wang Y., Wei S., Zhu J. (2023). Morphological, functional, compositional and transcriptional constraints shape the distinct venom profiles of the assassin bug *Sycanus croceovittatus*. Int. J. Biol. Macromol..

[57] Zhang G., Lu Z.Q., Jiang H., Asgari S. (2004). Negative regulation of prophenoloxidase (proPO) activation by a clip-domain serine proteinase homolog (SPH) from endoparasitoid venom. Insect Biochem. Mol. Biol..

[58] Thomas P., Asgari S. (2011). Inhibition of melanization by a parasitoid serine protease homolog venom protein requires both the clip and the non-catalytic protease-like domains. Insects.

[59] Wu C.Y., Huang J.M., Zhao Y.J., Xu Z.W., Zhu J.Y. (2020). Venom serine proteinase homolog of the ectoparasitoid *Scleroderma guani* impairs host phenoloxidase cascade. Toxicon.

[60] Price D.R.G., Bell H.A., Hinchliffe G., Fitches E., Weaver R., Gatehouse J.A. (2009). A venom metalloproteinase from the parasitic wasp *Eulophus pennicornis* is toxic towards its host, tomato moth (*Lacanobia oleracae*). Insect Mol. Biol..

[61] Lin Z., Cheng Y., Wang R.-J., Du J., Volovych O., Li J.C., Hu Y., Lu Z.Y., Lu Z., Zou Z. (2018). A metalloprotease homolog venom protein from a parasitoid wasp suppresses the toll pathway in host hemocytes. Front. Immunol..

[62] Colinet D., Dubuffet A., Cazes D., Moreau S., Drezen J.-M., Poirié M. (2009). A serpin from the parasitoid wasp *Leptopilina boulardi* targets the *Drosophila* phenoloxidase cascade. Dev. Comp. Immunol..

[63] Yan Z., Fang Q., Liu Y., Xiao S., Yang L., Wang F., An C., Werren J.H., Ye G. (2017). A Venom serpin splicing isoform of the endoparasitoid wasp *Pteromalus puparum* suppresses host prophenoloxidase cascade by forming complexes with host hemolymph proteinases. J. Biol. Chem..

[64] Yang L., Qiu L.M., Fang Q., Ye G.-Y. (2020). A venom protein, Kazal-type serine protease inhibitor, of ectoparasitoid *Pachycrepoideus vindemiae* inhibits the hemolymph melanization of host *Drosophila melanogaster*. Arch. Insect Biochem. Physiol..

[65] Zhou L., Wang R., Lin Z., Shi S., Chen C., Jiang H., Zou Z., Lu Z. (2023). Two venom serpins from the parasitoid wasp *Microplitis mediator* inhibit the host prophenoloxidase activation and antimicrobial peptide synthesis. Insect Biochem. Mol. Biol..

[66] Wang L., Fang Q., Zhu J., Wang F., Rean Akhtar Z., Ye G. (2012). Molecular cloning and functional study of calreticulin from a lepidopteran pest, *Pieris rapae*. Dev. Comp. Immunol..

[67] Siebert A.L., Wheeler D., Werren J.H. (2015). A new approach for investigating venom function applied to venom calreticulin in a parasitoid wasp. Toxicon.

[68] Yang L., Wang B., Qiu L., Wan B., Yang Y., Liu M., Wang F., Fang Q., Stanley D.W., Ye G. (2019). Functional characterization of a venom protein calreticulin in the ectoparasitoid *Pachycrepoideus vindemiae*. Insects.

[69] Kryukova N.A., Chertkova E.A., Semenova A.D., Glazachev Y.I., Slepneva I.A., Glupov V.V. (2015). Venom from the ectoparasitic wasp *Habrobracon hebetor* activates calcium-dependent degradation of *Galleria mellonella* larval hemocytes. Arch. Insect Biochem. Physiol..

[70] Wang L., Fang Q., Qian C., Wang F., Yu X.-Q., Ye G. (2013). Inhibition of host cell encapsulation through inhibiting immune gene expression by the parasitic wasp venom calreticulin. Insect Biochem. Mol. Biol..

[71] Ishii K., Adachi T., Hamamoto H., Sekimizu K. (2014). *Serratia marcescens* suppresses host cellular immunity via the production of an adhesion-inhibitory factor against immunosurveillance cells. J. Biol. Chem..

[72] Sláma K., Lukáš J. (2011). Myogenic nature of insect heartbeat and intestinal peristalsis, revealed by neuromuscular paralysis caused by the sting of a braconid wasp. J. Insect Physiol..

[73] Bai X., Yu K., Xiong S., Chen J., Yang Y., Ye X., Yao H., Wang F., Fang Q., Song Q. (2024). CRISPR/Cas9-mediated mutagenesis of the white gene in an ectoparasitic wasp, *Habrobracon hebetor*. Pest Manag. Sci..

