# Supplementary material for: Transcriptome Analysis Reveals the Venom Genes of the Ectoparasitoid Habrobracon hebetor (Hymenoptera: Braconidae)
Source: Insects. 2024 Jun 5;15(6):426. doi: 10.3390/insects15060426 (PMC11203415; doi:10.3390/insects15060426)
Supplement: Supplementary file 1 [file insects-15-00426-s001.zip › Table S1.pdf]

Table S1. Gene-specific primers used for qPCR analysis.

Gene name	Forward primer 5'-3′	Reverse primer 5'-3′
Calreticulin	CTACAACGATGCTGAGAAC	CAGGTCCGAACATAAGAAG
Cathepsin L	CACTGGTTGTCCTTATTGC	RCTGCTGTTGCTACTGTTG
Esterase	CTCAGCCAGCAGAATCAT	CACACCAGGACAGCATAT
Ion transport peptide-like	AACGGAAGAAGTCATAGGAG	GCAGATGGCGATGTTATTC
Lipase	CCTCGTCAATTCAAGCCTAT	CAGCATCCGAATCATCCA
Metalloproteinase 1	CCATTGAAGAACGCATAGAC	GAAGACACGCTACGCATA
Metalloproteinase 2	CTTCAATGACGAGGATTACG	AGTAGCAAGTCCAAGAGTAG
Paralytic protein	AAGGTTATGGCAGGAGAG	CAGGTTTGGATGGTAGTTG
Protein disulfide isomerase 1	TCACCGAGAGCAACAATG	ATCAGTCAGAGCCTTGGA
Serine protease 1	GACACTGCTGAATACATCTG	CTACCTGCCAATCTTACCA
Serine protease 2	AGCCTTCCTCTCATTACC	CCGAATTGGATGACTCTTG
Serine protease inhibitor 2	ACTGATGTAGAGACGATTGG	AGACAGGCACTCAAGATAC
UN1	CTAGCATTTGTGGTGGTG	CTGTTGGTGAGGAAGACT
18S RNA	AACTGGGGGCATTCGTATTG	CTTTCGCTGATGTTCGTCTTG
