# Supplementary material for: Transcriptome Analysis Reveals the Venom Genes of the Ectoparasitoid Habrobracon hebetor (Hymenoptera: Braconidae)
Source: Insects. 2024 Jun 5;15(6):426. doi: 10.3390/insects15060426 (PMC11203415; doi:10.3390/insects15060426)
Supplement: Supplementary file 1 [file insects-15-00426-s001.zip › Table S2.pdf]

Table S2: Fold change of venom genes expressed in the venom apparatus compared to the female residual body, determined using qPCR.

Gene name	Normalized expression value (Values shown on the Y-axis)		Fold change (V/RB)	Log2 fold change (V/RB)
	V	RB		
Paralytic protein	0.8950000	0.0003383	2645.32	11.37
Metalloproteinase 1	0.0480667	0.0000216	2228.75	11.12
Lipase	0.1070000	0.0000547	1957.32	10.93
Protein disulfide isomerase 1	0.0050900	0.0001870	27.22	4.77
Calreticulin	0.0231667	0.0010090	22.96	4.52
Ion transport peptide-like	0.0003360	0.0000002	1500.00	10.55
Metalloproteinase 2	0.0269333	0.0001917	140.52	7.13
Serine protease 1	0.0020800	0.0000042	500.00	8.97
Esterase	0.0069667	0.0001917	36.35	5.18
Serine protease 2	0.0041833	0.0000066	632.56	9.31
Serine protease inhibitor 2	0.0016567	0.0004783	3.46	1.79
Cathepsin L	0.0252667	0.0048867	5.17	2.37
UN1	0.0301667	0.0014900	20.25	4.34

V, venom apparatus; RB, residual body of female adult, female adult body deprived of venom apparatus.
